# Training Standards Statements of Family Medicine Postgraduate Training – A Review of Existing Documents Worldwide

**DOI:** 10.1371/journal.pone.0159906

**Published:** 2016-07-26

**Authors:** Elisabeth Flum, Sarah Berger, Joachim Szecsenyi, Sabine Marquard, Jost Steinhaeuser

**Affiliations:** 1 Department of General Practice and Health Services Research, University Hospital Heidelberg, Heidelberg, Germany; 2 Institute of Family Medicine, University Hospital Schleswig-Holstein, Campus Luebeck, Luebeck, Germany; University of South Australia, AUSTRALIA

## Abstract

**Introduction:**

For the effective and safe management of complex care needs for patients in community settings, high quality family medicine (FM) training programmes are needed. In less primary care oriented countries, training standards statements for FM postgraduate training are less commonly found. The aim of this study was to review international training standards statements in FM postgraduate training and to catalogue these statements to be used as a best practice standard guide for FM training programs in Germany.

**Materials and Methods:**

A structured three-tiered search was performed: a systematic literature search in MEDLINE^®^; a search of international indicator databases; and a search in grey literature, consisting of a survey of international experts and a search in “Google (Scholar)”. From all identified documents, training standards statements were extracted, translated and summarized into categories referring to the same quality aspect.

**Results:**

The search strategy revealed 25 relevant documents (MEDLINE^®^ n = 15, databases n = 2, experts n = 7, “Google” n = 1), containing 337 training standards statements. These were summarized into 80 statements. They covered structure quality (n = 35); process quality (n = 43); and two training standards statements referred to outcome quality (n = 2).

**Conclusion:**

A broad range of internationally sourced training standards statements for FM postgraduate training could be identified from countries with well-established primary care systems. Only few statements internationally referred to outcome quality, expressing the difficulty in assessing outcome. The resulting inventory of training standards statements for FM postgraduate training can serve as a resource for institutions seeking to formalise and systematise FM training at regional or national levels.

## Introduction

To improve health care quality, the American Institute of Medicine recommended the improvement of education for health care professionals [1,2]. Family medicine (FM) is a complex medical specialty requiring a broad spectrum of clinical as well as non-clinical competencies and skills [3,4]. For the effective and safe management of complex care, high quality FM training programmes are needed [5]. Training standards statements for FM training programmes are also essential. These can be found in training documents from health care systems with strong primary care services as, for example, the United Kingdom (UK) or Canada. In 2013, the WONCA working party on education defined standards for FM postgraduate training by adapting the *Postgraduate Medical Education World Federation for Medical Education (WFME) Global Standards for Quality Improvement* [6]. A set of global standards consisting of nine areas including, for example, evaluation training process, assessment of trainees and educational resources was developed [7]. Besides definition of training standards, assessment and supervision of training outcomes were deemed also necessary [8].

In Germany, a country with a less developed primary care system, none of these training standards statements have yet been implemented into the mandatory (five-year) FM postgraduate training. Despite formal standards from the German Medical Council, nationally no specific training standards and no quality criteria are defined [9,10]. One exception is an independent state level initiative, the Verbundweiterbildung^*plus*^ Baden-Wuerttemberg (Verbundweiterbildung^*plus*^), which offers a competence-based postgraduate training in FM within purpose specific training posts [11]. It includes continuing and structured rotation through hospital and ambulatory care settings as well as regular education seminars [12,13]. Following recognition by the German College of General Practitioners and Family Physicians (DEGAM), other federal states in Germany have also begun to develop similar training programs for FM. This is raising the impetus to formalise training standards based on a national catalogue from the German College of General Practitioners and Family Physicians (DEGAM-Verbundweiterbildung^*plus*^) [14].

Aim of this study was to review international training standards statements for FM training programmes and to catalogue these statements for use in FM postgraduate training in Germany.

## Materials and Methods

The structured search strategy for internationally established training standards statements and quality criteria in FM postgraduate training programs had 3 elements: a systematic MEDLINE^®^ literature search; a search of international indicator databases; and a search in “grey literature”, consisting of a survey of international experts and a search in “Google (Scholar)”.

An exploratory search was performed to define a model for the systematic literature search. Various strategies were tested in MEDLINE^®^ with different combinations of Medical Subject Headings (MeSH)-terms and free text. The results of these tests were used to optimise and specify the final search strategy. Table 1 shows the final search strategy.

**Table 1:** Final search strategy.

family medicine	postgraduate training	quality criteria
(“general practice” [mh]	(“education, medical, graduate”	(“quality indicators, health care”
OR	[mh] OR	[mh] OR
“general pract*”	“graduate medical education”	“quality indicator*”
OR	OR	OR
“family practice” [mh]	“internship and residency” [mh]	“quality assurance, health care”
OR	OR	[mh] OR
“family practice”	internship	“quality assurance”
OR	OR	OR
“primary health care” [mh]	residency	“accreditation” [mh]
OR	OR	OR
“primary health care”	“curriculum” [mh]	“accreditation”
OR	OR	OR
“physicians, primary care” [mh]	“curriculum”	“benchmarking” [mh]
OR	OR	OR
“general practitioners” [mh]	“vocational training”	benchmarking
OR	OR	OR
“general medicine”	“postgraduate education”	“clinical audit” [mh]
OR	OR	OR
“family medicine”)	“postgraduate training”	“clinical audit”
	OR	OR
	“special* training”	“quality measures”
	OR	OR
	“competency based education”)	“quality criteria”)
Filters: Publication date from 1967/01/01 to 2014/04/10, English, German		
**#1 AND**	**#2 AND**	**#3**

To differentiate between relevant and irrelevant manuscripts, inclusion and exclusion criteria were pre-defined. Inclusion criteria were specified as follows: publication referred to FM postgraduate training and defined training standards covering different aspects such as training in hospital or practice, curriculum and educational seminars. Furthermore, training standards regarding assessment of knowledge and competencies as well as the trainees’ and trainers’ perspective were included. Exclusion criteria were defined as follows: publication did not refer to FM postgraduate training or no quality standards were specifically named. Publications were also excluded, if only an abstract was available, no full text in English or German language was obtainable or if the chosen time period was not met.

### Systematic literature search and search of indicator databases

The systematic literature search was carried out in the database MEDLINE^®^ for the time period from 10-04-2014 back to 01-01-1967. Four researchers in two pairs, screening independently from each other, assessed title and abstract according to the pre-defined inclusion and exclusion criteria. Results were compared and discussed by the pairs of researchers and a consensus version created. If discussion did not result in a consensus, the publication was included in the second round, where full text screening occurred. If available, all publications identified as potentially relevant were ordered in full text. The full text screening process was carried out in the same way as the title and abstract screening by the pairs of researchers. All publications identified as relevant after the full text screening were included in the study.

International indicator databases were identified by a list of international databases, which was obtained from the Institute for Applied Quality Improvement and Research in Healthcare (AQUA-Institute), a renowned institution in Germany for health care quality [15]. An indicator database is a (commonly freely) accessible website that contains collections of quality standards. The list of the indicator databases used is available from the corresponding author. Two researchers searched the databases independently from each other according to the predefined inclusion and exclusion criteria. As with the literature review, results were compared and discussed by researchers resulting in a consensus version. If a consensus was not possible, the document of the database was included in the study for subsequent further evaluation. Finally, a list with relevant documents of databases was developed.

### Search in grey literature

All member countries of The World Organization of National Colleges, Academies and Academic Associations of General Practitioners / Family Physicians (WONCA) Europe were contacted via email to the international chairman or president indicated by the official website of WONCA Europe [16]. The member countries were asked to name experts in FM postgraduate training, who could potentially provide specific documents containing training standards. Furthermore, the researchers contacted experts in FM postgraduate training using personal networks from previous international projects [17]. All experts were asked to provide documents regarding training standards in FM postgraduate training programs. Furthermore, unstructured hand searches in the searching machine “Google” and “Google Scholar” were performed by the research team, using the terms defined for the systematic literature search. Again, all identified documents were reviewed according to the inclusion and exclusion criteria and discussed by two independent researchers, resulting in a final list of relevant documents. All correspondence with the experts was done via email.

### Standardised extraction of data

A structured template for the extraction and preparation of the identified training standards was obtained from the AQUA-Institute [15] and adapted to the FM training context. The template contained for example the following items: specification, original wording, domain and reference list; the template is available from the corresponding author. The identified training standards were translated, catalogued and summarized into categories referring to the same quality aspect by a researcher. This was carried out by the research team based on the checklist developed by the DEGAM-Verbundweiterbildung^*plus*^ [14]. The results were reviewed and discussed by the research team resulting in a final consensus version. Similar training standards referring to the same aspect were summarized as variations into one template to ensure comprehensibility and to reduce volume. The training standards were assigned to three categories: structure, process and outcome quality according to Donabedian [18].

## Results

### Systematic literature search and indicator databases

The systematic literature was performed on 10-04-2014 in the database MEDLINE^®^ and resulted in 2.016 hits. Screening of title- and abstracts excluded 1.918 publications, leaving 98 publications for full text screening. From all full texts, 83 were excluded, 15 relevant publications were included.

The list of international indicator databases provided by the AQUA-institute contained 73 databases. From 20-03-2014 to 23-03-2014, the search of the databases was performed according to the strategy described in the Methods section; two relevant documents were included. A 2013 WONCA document recommended by one of the reviewers was also subsequently included [7].

### Grey literature

**Fig 1:**
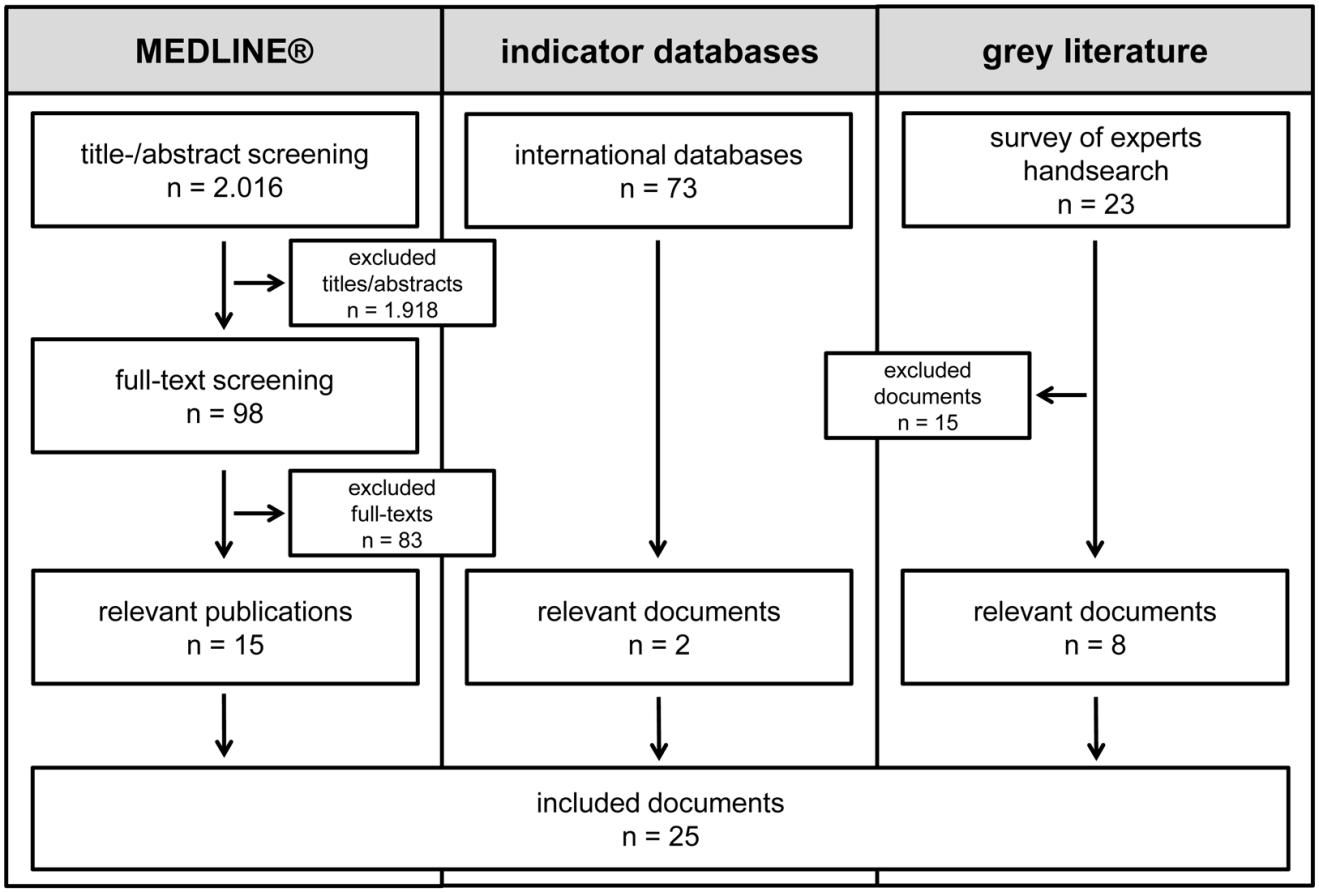
Results of the search strategy.

From 09-01-2014 to 30-05-2014, all 39 member countries of WONCA Europe and the six experts in FM postgraduate training personally known to the researchers were contacted via email. All in all, eight experts answered. Luxemburg and the Netherlands could only provide training standards in the respective language of the country and not in English or German. Switzerland was not able to provide documents specific for FM. Due to the predefined inclusion and exclusion criteria, these documents were not included. In total, 23 documents were reviewed by the researchers: Australia and New Zealand (n = 8), European Academy of Teachers in General Practice / Family Medicine (EURACT, n = 7), the UK (n = 2) and the United States (USA, n = 5). Included according to the predefined criteria were: Australia (n = 1), New Zealand (n = 1), EURACT (n = 2), UK (n = 2) and USA (n = 1). From the hand search in “Google”, one relevant Canadian document was included. The S1 Table presents the results of the survey of experts. All in all eight relevant documents were included into the study. Fig 1 shows the results of the systematic search strategy. Table 2 shows all included documents.

**Table 2:** Documents included in the analysis.

No	Country	Author	Titel	Date	Training Standard Statements	Identification Method
1*	Woldwide	WONCA	WONCA Global Standards for Postgraduate Family Medicine Education	2013	39	expert
2	Europe	EURACT	EURACT Statement on Selection of General Practice/ Family Medicine (GP/FM) Trainers/ Practices and Implementation of Specialist Training in GP/FM	2012	26	expert
3	Europe	EURACT	EURACT Statement on Hospital Posts used for Training in General Practice/Family Medicine	2013	16	expert
4	Australia	Australian Medical Council	Standards for Assessment and Accreditation of Specialist Medical Education Programs and Professional Development Programs	2010	54	expert
5	Canada	The College of Family Physicians of Canada	Standards for the Assessment of Non- Canadian Postgraduate Family Medicine Education Programs	-----	39	hand search
6	New Zealand	Medical Council of New Zealand	Additional criteria for Assessment of Specialist Medical Education Programmes and Professional Development Programmes	2013	2	expert
7	Singapur	Wong TY et al.	Postgraduate Family Medicine Training in Singapore A New Way Forward	2012	13	MEDLINE^®^
8	UK	General Medical Council (NHS)	Standards for curricula and assessment systems	2010	17	Indicator database
9	UK	General Medical Council (NHS)	The Trainee Doctor- Foundation and specialty, including GP training	2011	23	Indicator database
10	UK	Kibble S et al.	The application process for general practitioner trainers in United Kingdom deaneries: similarities and differences	2009	0	MEDLINE^®^
11	UK	Irvine D et al.	The accreditation of vocational training programmes- results of a pilot survey	1974	3	MEDLINE^®^
12	UK	NHS, East Midlands Healthcare Workforce Deanery	The East Midlands Quality Standards for Specialty Schools	2008	35	expert
13	UK	NHS, Health Education East Midlands	GP Specialty Training Approval Document	-----	38	expert
14	UK	Smith VC et al.	Describing the learning climate of general practice training: the learner’s perspective	2009	0	MEDLINE^®^
15	USA	Accreditation Council for Graduate Medical Education	Program Requirements for Graduate Medical Education in Family Medicine	2014	36	expert
16	USA	Catinella AP et al.	The Utah Rural Residency Study: A Blueprint for Evaluating Potential Sites for Development of a 4-4-4 Family Practice Residency Program in a Rural Community	2003	1	MEDLINE^®^
17	USA	Eiff MP et al.	A Model for a Standardized National Family Medicine Graduate Survey	2009	0	MEDLINE^®^
18	USA	Geyman JP et al.	Prevention of Complications in Initial Development of Family Practice Residency Programs	1977	9	MEDLINE^®^
19	USA	Girard DE et al.	A comparison study of career satisfaction and emotional states between primary care and specialty residents	2006	0	MEDLINE^®^
20	USA	Green LA et al.	Preparing the Personal Physician for Practice: Changing Family Medicine Residency Training to Enable New Model Practice	2007	5	MEDLINE^®^
21	USA	Lurie SJ et al.	Measurement of the General Competencies of the Accreditation Council for Graduate Medical Education: A systematic review	2009	0	MEDLINE^®^
22	USA	Mc Cann WJ et al.	Hidden in Plain Sight: Residency Coordinator’s Social Support of Residents in Family Medicine Residency Programs	2011	0	MEDLINE^®^
23	USA	Musick DW et al.	A Conceptual Model for Program Evaluation in Graduate Medical Education	2006	1	MEDLINE^®^
24	USA	Rieselbach RE et al.	Academic Medicine: A Key Partner in Strengthening the Primary Care Infrastructure Via Teaching Health Centers	2013	16	MEDLINE^®^
25	USA	Schuhmacher DJ et al.	Perspective: Beyond Counting Hours: The Importance of Supervision, Professionalism, Transitions of Care, and Workload in Residency Training	2012	3	MEDLINE^®^
26	USA	Shaugnessy AF et al.	EPA in Family Medicine	2013	0	MEDLINE^®^

Abbreviations: EURACT = European Academy of Teachers in General Practice / Family Medicine; FM = family medicine; GP = General Practitioner; NHS = National Health Service; UK = United Kingdom; USA = United States of America

*This document was included after the original analysis based on the recommendation of a reviewer of the manuscript

### Standardised extraction of data

From all identified documents, 337 training standards statements for FM postgraduate training were extracted and translated. Repeated statements referring to the same training standard were summarized into one template, resulting in 80 categories. Some templates contained up to 20 statements covering the same aspects. For example, the category “supervision of trainees” was named several times. All identified and summarized training standard statements were classified into three categories: structure, process and outcome quality. Table 3 shows all identified training standards statements related to the category of structure quality (n = 35). Training standards statements related to the category of process quality (n = 43) are displayed in Table 4. Table 5 lists all training standards statements related to the category of outcome quality (n = 2).

**Table 3:** Training standards statements related to structural quality of postgraduate training (n = 35).

Structure Quality: The FM postgraduate training program…		
No	Statement	Source according to Table 2
1	… is family medicine directed	1,5,15
2	… cooperates with the medical faculty of a university	1,5
3	… has a letter of agreement between the program and each collaborating partner	15
4	… has information about the program made publicly accessible	4,11,12
5	… has a policy on trainee involvement integrated into the governance of the program	1,4,5
6	… has appropriate staff planning in training posts	1,5,9,15,18
7	… has transparent policies and defines responsible persons at each stage of training	1,4,5,12,15
8	… has a sponsoring institution assuming ultimate responsibility for the program	1,5,15,18,24
9	… defines requirements for trainees	3,4
10	… has a process for selection of trainees	1,4,5,15
11	… has a policy for recognition and assessment of international medical graduates	6
12	… has a policy for resident transfers from other training programs	15
13	… has a policy for recognition of training credits from other training sites or other countries	1,5
14	… defines requirements for trainers	12
15	… has written regulatory framework of the program	1,3,4,5,9,15,18, 24
16	… defines requirements for examiners	4,8
17	… has a defined and documented assessment system	4,8
18	… offers appropriate working time models	4,12
19	… provides clinical rotations to require specific competencies for FM	3,7,24
20	… has a policy for signing a training agreement at the start of each post	12
21	… has defined overall goals of training (e.g. task-based outcomes)	4,8
22	… provides role-modelling for clinical governance	2,13
23	… provides the trainees with the educational opportunities defined in the curriculum	2,3,4,8,12,13,16
24	… provides regular educational meetings	12
25	… provides training in cultural competence	6,24
26	… provides training in effective use of health information technology	1,5,13,20,24
27	… provides adequate education facilities	1,2,3,5,9,13,15
28	… provides a room for trainees‘ consultations in teaching practices	2,13
29	… provides working conditions in interprofessional teams	4
30	… has a policy for dealing with routine activities with no educational value to the trainee	12,13,15
31	… maintains critical incident reports	2,13
32	… offers career advice	9,12,24
33	… ensures a counseling service	1,4,5,9,18,24
34	… provides safe working environment	2,13
35	… complies with national safety legislation	2,12,13

**Table 4:** Training standards statements related to process quality of postgraduate training (n = 43).

Process Quality: The FM postgraduate training program…		
No	Statement	Source according to Table 2
36	… gives an induction to the trainee at the start of every post	2,9,12,13
37	… defines a rotation schedule for training posts	18,24
38	… offers working patterns appropriate for learning	1,5,9,13,15
39	… provides an introduction to the curriculum	8
40	… implements and uses the curriculum	2,7,18
41	… ensures educational progress is defined for each stage by the curriculum	1,4,5,7
42	… performs regular curriculum review and updating	1,4,5,8
43	… contributes to continuity from undergraduate to postgraduate medical training	1,4,5
44	… names an assigned educational supervisor for every trainee	1,2,3,4,5,8,9,12,13,15,18,24,25
45	… offers accredited teachers’ courses	1,2,3,4,5,8,12,13
46	… performs an assessment of trainees‘ educational needs (learning needs analysis)	2,3,7,13
47	… defines a personal learning plan for every trainee	2,3,8
48	… encourages the trainee to maintain a personal logbook / training portfolio	9,12,13
49	… performs trainer-observed consultations	2,12
50	… gives regular constructive feedback about trainees performance	1,4,5,9,12,15,24
51	… identifies early trainees who underperform	4,9,13
52	… notifies any significant change in the trainees’ training process	13
53	… addresses immediately any concerns about patient safety arising from the trainees	9,13,15
54	… offers protected teaching and learning time	1,2,3,4,5,11,13, 15
55	… performs a diversity of learning methods	2,8,9
56	… encourages trainees to teach each other	18,24,25
57	… exposes trainees to academic opportunities and research	1,4,5,12,15
58	… ensures trainees participation in regular team meetings	2,13
59	… holds regular meetings of clinical competency committee	4,7,15
60	… ensures completed resuscitation training by every trainee	12
61	… provides training in shared decision making	13
62	… provides training in interdisciplinary teams	1,2,3,5,11,12,13,15,20,24
63	… provides training in structured hand-overs for continuity of care and patient safety	9,15,18,25
64	… provides training in generic professional skills as team leadership and conflict management	7,12,13,24
65	… provides training in critical appraisal of literature, scientific data and evidence-based medicine	1,4,5,7,15
66	… provides training in application for study leave and funding	12
67	… provides training in quality management	7,9,24
68	… provides training in the use of assessment tools	12
69	… has a defined assessment system	1,2,4,5,7,8,12, 15,20
70	… uses multifaceted assessment methods	1,4,5,7,8
71	… documents assessment and the trainee’s progress	4,8
72	… performs program evaluation and quality assurance	1,2,3,4,5,8,9,13,15
73	… performs an audit cycle for quality improvement	2,13
74	… selects and regularly reselects training posts	1,3,5
75	… carries out satisfaction surveys on trainees and trainers	1,4,5,12,13
76	… carries out patient satisfaction surveys	2,13,20
77	… includes lay people and patients in training and assessment processes	8
78	… informs patients about FM training posts	2,13

**Table 5:** Training standards statements related to outcome quality of postgraduate training (n = 2).

Outcome Quality: The FM postgraduate training program…		
No	Statement	Source according to Table 2
79	… defines graduate outcomes addressing the roles in FM, technical and clinical expertise	1,5,15
80	… documents successful completion of training	4

## Discussion

### What is known and what is new

Although training standards statements are found in FM postgraduate training documents, this is the first attempt to create an inventory of exemplary statements. Our final set of internationally sourced training standards statements for FM postgraduate training programs contained 80 statements, resulting in detailed and elaborate elements. This catalogue can serve as a best practice standard guide for FM training programmes in Germany. It is also a useful resource for other nations in the process of formalising and systematising their FM training programmes.

Most of the identified internationally used training standards statements fell into the category of process quality, for example “learning need analyses” or “trainer-observed consultations”. In second place came the training standards statements for structure quality, for example “cooperation with a medical faculty of a university” or “providing of safe working environment”. Only two training standards statements were found for the category of outcome quality, reflecting the fact that medical education is still more structure- and process- and less outcome-based, as outcomes are more difficult to assess and less under control of physicians [8]. There is a need for comprehensive programme evaluation processes [9]. Several training standards statements referred to non-technical skills as “shared decision making” or “generic professional skills such as team leadership or conflict management”, which are also important to enhance collaboration with patients and other members of the health care team and reduce medical errors from trainee doctors [19].

Several identified training standards statements, for example, requirements for training, selection process of trainees or assessment methods, relating to the regulatory framework of the nation, were specific to the national context of the respective country and its health care system making it difficult to adapt them for the German setting. Furthermore, many identified training standards statements described very generic aspects, for example: “The trainee must achieve knowledge of scientific basis and methods of family medicine and (…) become familiar with evidence-based medicine and critical clinical decision making.” [20]. Generic statements have the potential to create difficulties in assessment.

For adaptation to other countries regulatory frameworks and health care systems, a validation process, for example, a group facilitating technique is necessary [21]. The identified training standards statements for FM postgraduate training programmes rarely indicated classification or validation processes. Gaining access to such information would be very useful for nations seeking to formalise and systematise their FM training programmes.

### Strength of this study

The strength of this study is that it provides a first comprehensive review of international documents with training standards statements for FM postgraduate training programs. Our extensive search strategy was intended to cover all relevant aspects of postgraduate training in FM. Most of the relevant documents were found via hand searching in grey literature or provided by experts, meaning that the identified documents may possibly not be representative worldwide. This underlines the necessity for more publicly available online resources to be shared in the interests of facilitating collaboration, benchmarking and best practice standards for FM training internationally.

### Limitations of the study

The response rate of the contacted WONCA Europe-member countries was low (3 countries out of 39 responded). Interestingly, none of the participating experts referred to the “WONCA Global Standards for Postgraduate Family Medicine Education” [7]. This reference was not included in the analysis until one reviewer of the manuscript recommended it. As the document is to a large extent congruent with the *Standards for the Assessment of Non-Canadian Postgraduate Family Medicine Education Programs*, which was included in the original analysis, the results did not differ after inclusion of the WONCA standards. Not all identified documents were available in English or German and therefore had to be excluded for this reason, adding a further limitation to the data available for analysis.

### Future developments

Countries with a primary health care system that is placed centrally in the health care system have elaborately defined training standards for FM postgraduate training programs, which can be a resource for other nations. The final set of internationally acceptable training standards statements produced in this process could serve as a best practice guide for other countries that are in the process of formalising and systematising FM training. For Germany, the next step will be the selection and cultural adaptation process of the identified training standards statements by an expert panel using a modified RAND/UCLA method [22]. Afterwards, a pilot study will be undertaken to test validity and reliability of the training standards statements for FM postgraduate training programs.

## Conclusion

An inventory of 80 training standards statements have been identified and collated from 26 FM training standards documents worldwide—consisting of 35 structure, 43 process, and 2 outcome statements. These training standards statements could be of value to other countries in the process of developing FM postgraduate training programs.

## Supporting Information

S1 Table Results of the survey of experts. Abbreviations: EURACT = European Academy of Teachers in General Practice / Family Medicine; FM = family medicine.
(DOCX)
