# Supplementary material for: Training Standards Statements of Family Medicine Postgraduate Training – A Review of Existing Documents Worldwide
Source: PLoS One. 2016 Jul 26;11(7):e0159906. doi: 10.1371/journal.pone.0159906 (PMC4961452; doi:10.1371/journal.pone.0159906)
Supplement: S1 Table — Abbreviations: EURACT = European Academy of Teachers in General Practice / Family Medicine; FM = family medicine. (DOCX) [file pone.0159906.s001.docx]

**S1 Table: Results of the survey of experts**

| **Countries** | **Contact** | **Identified documents** | **Included documents** |
| --- | --- | --- | --- |
| EURACT | Personal contact | n=7 [in English] | n=2 |
| Australia and New Zealand | Personal contact | n=8 [in English] | n=2 |
| Luxemburg | Official contact by WONCA | Document not in English or German | n=0 |
| Netherlands | Official contact by WONCA | Document not in English or German | n=0 |
| Switzerland | Official contact by WONCA | Document not specific for FM | n=0 |
| United Kingdom | Personal contact | n=2 [in English] | n=2 |
| United States | Personal contact | n=5 [in English] | n=1 |

Abbreviations: EURACT= European Academy of Teachers in General Practice / Family Medicine; FM=family medicine
